# Interleukin 6 Function in the Skin and Isolated Keratinocytes Is Modulated by Hyperglycemia

**DOI:** 10.1155/2019/5087847

**Published:** 2019-04-03

**Authors:** Eric G. Lee, Lerin R. Luckett-Chastain, Kaitlin N. Calhoun, Benjamin Frempah, Anja Bastian, Randle M. Gallucci

**Affiliations:** Department of Pharmaceutical Science, College of Pharmacy, University of Oklahoma Health Sciences Center, P.O. Box 26901, Oklahoma City, OK 73126, USA

## Abstract

Diabetes currently affects over twenty-five million Americans. Annual health care cost of diabetes exceeds $254 billion and is associated with a distinct set of diabetic complications that include delayed wound healing and diabetic ulcers. Interleukin 6 (IL-6) plays an important role in wound healing and is known to be elevated in the serum of both type I and type II diabetes patients. This study assesses the expression and function of IL-6 in the hyperglycemic epidermis and keratinocyte culture. Streptozotocin-treated mice were wounded six weeks after induction of hyperglycemia. Wound closure, protein, and mRNA expression were assessed up to 13 days of postwounding. Wound closure was delayed 4-5 days in hyperglycemic animals. Hyperglycemic wounds displayed greater IL-6 and IL-6R*α* protein expression at 1, 7, and 10 days of postwounding compared to euglycemic control. However, IL-6R*α* mRNA expression was reduced at all time points beyond day 1, while IL-6 mRNA expression did not significantly differ at any time point. SOCS3 mRNA expression was higher in the hyperglycemic skin at every time point. Imaging of fluorescent immunohistology also revealed significantly lower expression of SOCS3, but higher nuclear pSTAT3 in the epidermis of the hyperglycemic skin. Primary mouse keratinocytes cultured in high glucose for 7 days displayed 2-fold higher IL-6R*α* mRNA and higher rmIL-6-induced nuclear pSTAT3, but lower SOCS3 basal levels compared to normal glucose-cultured cells. Thus, it appears that delayed diabetic skin wound healing is associated with increased induction and expression of IL-6 and its receptor, but its function in epidermal keratinocytes may be impaired.

## 1. Introduction

Diabetes is an increasingly serious public health problem throughout the world. In the US, greater than 30 million people are diabetic with another 84 million considered prediabetic [1]. This translates to an annual health care cost that exceeds $245 billion, encompassing 25% of the total US health care spending [2]. Chronic nonhealing wounds such as diabetic ulcers are the leading cause of nontraumatic lower leg amputations [3]. This condition greatly affects the quality of life while contributing to an increased risk of mortality. While diabetes has been reported to alter numerous systems, including vascular and immune, efforts to establish the mechanisms by which diabetes alters wound healing have been lacking.

Diabetic ulcers are known to display defects in nearly all phases of the wound healing process. Normal wound healing is typically broken into four overlapping phases: (1) coagulation, (2) inflammation, (3) proliferation, and (4) remodeling (for review, see [4]). Interruption of any of these phases could potentially delay healing. A dysregulated inflammatory phase is an often reported deficiency found in diabetic wounds [5, 6] that is characterized by a late yet sustained inflammatory infiltrate [7]. Altered levels of proinflammatory cytokines such as TNF-*α*, IL-1, and IL-6 are also reported and appear to be regulated differently in the early [8] versus later chronic phases [9] of wound healing, with overexpression dominating the later phases.

While diabetes alters numerous aspects of wound healing, it is interesting that hyperglycemia seems to be an important modulator of wound healing, as healing improves if glucose levels are reduced [10]. Mouse keratinocytes [11] and fibroblasts isolated from the diabetic skin have an impaired proliferative response in high-glucose conditions [12], whereas Tenon's capsule fibroblasts show the opposite [13]. Hyperglycemia alone upregulates the expression of inflammatory cytokines such as TNF-*α*, IL-1, IL-6, and chemokines in monocytes [14, 15], epithelial cells [16], and plasma [17]. Hyperglycemia also induces inflammatory signaling through activation of both the NF-*κ*B and AP-1 pathways in vascular smooth muscle [18], monocytes [19], mesangial cells [20], hepatocytes [21], and endothelial cells [21]. IL-6 is an important proinflammatory cytokine associated with wound healing [22, 23] and prominently expressed in serum during numerous diabetic pathologies, including retinopathy [24], cardiovascular disease [25], immunosuppression [26], and renal disease [27]. While both diabetes and hyperglycemia increase circulating IL-6 levels [17, 28], it is unclear whether altered IL-6 is a cause or an effect of diabetes. However, it is known that IL-6 alters the activity of numerous types of cells including decreasing insulin sensitivity in the liver and adipocytes possibly through crosstalk between the IL-6 and insulin receptors via suppressors of cytokine signaling (SOCS) proteins [29].

Dysregulation of IL-6 function is a common observation in various diabetic complications. Given the association of nonhealing diabetic ulcers with inflammation, the altered levels of IL-6 observed during diabetes/hyperglycemia, and the close relationship of this cytokine with skin healing, it is likely that IL-6 and/or its receptor may play important roles in diabetic wound healing. It has been shown to be variably modulated in animal [8, 30] and human [31] chronic wounds.

Herein, evidence is provided to suggest that delays in diabetic wound healing may be associated with increased IL-6, IL-6R*α* expression, and STAT3 activation, yet lower SOCS3 expression in the skin. Further, hyperglycemia itself can reproduce these effects in keratinocyte cultures where IL-6-induced p-STAT3 levels are increased compared to control. Given these observations, IL-6 induction and IL-6R function would appear to be distinctly dysregulated during diabetes and hyperglycemia, possibly contributing to delays in healing by affecting the epidermal response to this cytokine.

## 2. Methods

### 2.1. Streptozotocin-Induced Hyperglycemia

Male C57BL/6 mice (age: 6-8 weeks) were purchased from Jackson Laboratory (Bar Harbor, Maine). Hyperglycemia was induced by repeated (once per day for five days) intraperitoneal injection of streptozotocin (STZ, 50 mg/kg, dissolved in citrate buffer) [32]. Following one week recovery from STZ treatment, blood glucose levels were analyzed. Mice with blood glucose levels greater than 350 mg/dl for four weeks were considered hyperglycemic and used as experimental animals. Age-matched mice were utilized as experimental controls.

### 2.2. In Vivo Wounding

Following four weeks of consistent hyperglycemia, both hyperglycemic and age-matched control mice were anesthetized with isoflurane (Baxter, Deerfield, IL) and an area (~10 cm^2^) of the skin just posterior to the skull was denuded by clippers then swabbed with Betadine® (Purdue Fredrick Co., Norwalk, CT) and 70% ethanol three times. Four 4 mm punch biopsies were performed on the shaved area utilizing sterile single-use biopsy punches. Wound healing was assessed daily for fifteen days by measuring wound closure from photographs using ImageJ (NIH Image; https://rsbweb.nih.gov/ij/). For comparison, a 4 mm circular paper biopsy reference was included in each wound photograph as a means of standardizing photos. Wound tissue was collected on days 0, 1, 3, 7, and 10 for RNA, protein, and histological analysis. Wound tissue was immediately homogenized in TRI Reagent (Molecular Resource Center, Cincinnati, OH) for RNA or RIPA buffer plus PMSF and protease inhibitor (cat. no. P8340; Sigma) for protein isolation or fixed in 10% buffered formalin for histology. Sections of these samples were stained with hematoxylin/eosin or stained for SOCS3 (ab76315, Abcam, Cambridge, UK) and pSTAT3 (ab76315, Abcam) followed by anti-rabbit Alexa Fluor-488 (Life Technologies, Carlsbad, CA) and nuclear staining with DAPI.

### 2.3. Isolation and Culture of Primary Keratinocytes

For isolation of neonatal mouse epidermal keratinocytes (MEKn), C57 neonatal mice (<72 hours) were euthanized by decapitation and the skin was removed. The skin was incubated overnight in PBS with dispase (25 U/ml) at 4°C. Using sterile forceps, the epidermis was separated from the dermis and incubated in 0.25% trypsin at 37°C for 3-5 min, followed by neutralization with EpiLife medium + 0.045 mM calcium chloride + 1 *μ*l/ml EDGS (EpiLife® Defined Growth Supplement (EDGS; Life Technologies)), straining through gauze and centrifugation at 200 g for 5-10 min. Cells were immediately seeded on collagen I-coated plates in EpiLife + EDGS medium. The following day, media was switched to EpiLife + EDGS medium containing normal glucose (5 mM), high glucose (25 mM), or mannitol (5 mM glucose + 20 mM mannitol) and cultured 37°C and 5% CO_2_ and 95% room air and maintained for 7 days. After 7 days, cells were treated with recombinant mouse (rm) IL-6 (BioLegend, San Diego, CA) and immunostained or lysate collected for Western blotting. For pSTAT3, immunostaining cells were seeded in a collagen I-coated 96-well plate after isolation. Cells were immunostained for pSTAT3 (Y705, Abcam ab76315) or SOCS3 (ab76315, Abcam, Cambridge, UK), followed by anti-rabbit Alexa Fluor-488 (Life Technologies) and DAPI (2.5 *μ*g/ml). Images were obtained with Operetta High-Content Imaging System (PerkinElmer, Waltham, MA) and analyzed with Columbus (v2.6.0, PerkinElmer). The percentage of nuclear positive cells was determined by using a threshold value for pSTAT3 or SOCS3 staining intensity that colocalized with DAPI. Western blotting was performed for total STAT3 (#9139, Cell Signaling, Danvers, MA) and for pSTAT3 (Y705) (#9145, Cell Signaling) as described previously (Bastian et al., 2017).

### 2.4. Real-Time Quantitative RT-PCR

Total RNA from the mouse skin or MEKn was prepared and cDNA synthesized as described previously [33]. Primers for mouse genes were generated from GenBank sequences, synthesized by Invitrogen, and are as follows:

- Socs3-5′GGACCAGCGCCACTTCTTCAC; Socs3-3′TACTGGTCCAGGAACTCCCGA
- IL6-5′TCAATTCCAGAAACCGCTATGA; IL6-3′CACCAGCATCAGTCCCAAGA
- IL6R-5′TGCCAACCTTGTGGTATCAGCC; IL6R-3′TGAAGACACAGAGAAGCACC

Quantitative RT-PCR (qPCR) was performed on an ABI PRISM 7000 SDS, utilizing AnaSpec SYBR Green MasterMix (Fremont, CA) according to the manufacturer's instructions. Quantitative values of genes of interest were normalized based on 28s rRNA content using the ddCt method [34].

### 2.5. ELISA Analysis

IL-6 and IL-6R*α* protein levels (eBioscience, San Diego, CA) from skin sample extracts were measured via ELISA and were performed according to the manufacturer's instructions.

### 2.6. Statistical Analysis

All experiments were replicated at least three times, and representative findings are shown. Statistical significance was determined by the nonparametric Mann-Whitney test. In all statistical comparisons, a *p* value of <0.05 was used to indicate a significant difference.

## 3. Results

### 3.1. STZ-Induced Hyperglycemic Mice Display Delayed Wound Closure

**Figure 1:**
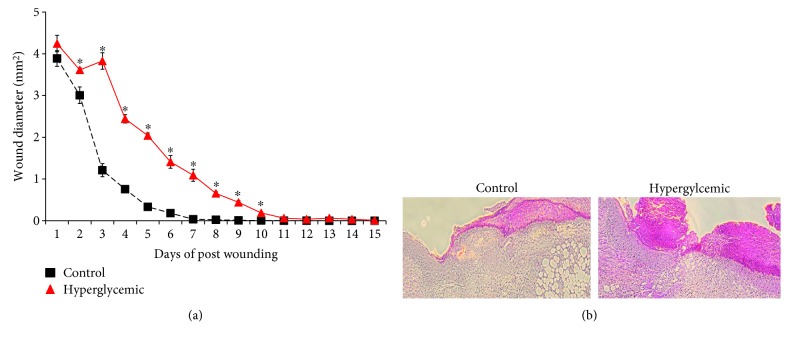
Delayed wound closure in hyperglycemic mice. Four- to six-week-old male C57BL/6 mice were treated with STZ once per day for 5 days to initiate diabetic hyperglycemia. Six weeks of postinduction, four 4 mm diameter full-thickness wounds were produced and photographed daily for 15 days. (a) Wound images were analyzed and wound diameter determined via ImageJ. Data are expressed as means ± SE (*n* = 10). ^∗^Significantly different (*p* < 0.05) from euglycemic C57 (control) mice. (b) On day three postwounding, wounds were collected and hematoxylin/eosin-stained sections were assessed for differences in wound healing using a Leica DM400B microscope at 100x magnification. Slides shown are representative of ten samples per treatment group.

To assess wound healing, diabetic mice and nondiabetic control mice (*n* = 10/group) were wounded and wound closure was measured for fifteen consecutive days. While splinted wounding [35] has been used in numerous diabetes studies, the nonsplinted model that was used as inflammation was the primary focus of the study and not epithelialization. Indeed, mechanical tension can influence cytokine expression in dermal fibroblasts [36]. Regardless, hyperglycemic animals displayed significantly delayed wound closure from day 2 through 15 but the delay was predominately associated with days 1-4 (Figure 1(a)). Complete wound closure occurred approximately four days later in hyperglycemic animals compared to control animals (day 7 vs. day 11). Histological examination showed that hyperglycemic wounds displayed less keratinocyte migration in the epidermal leading edges and decreased granulation tissue formation (Figure 1(b)) agreeing with previous reports [37, 38].

### 3.2. IL-6 and IL-6R*α* Are Altered in Diabetic Wounds

**Figure 2:**
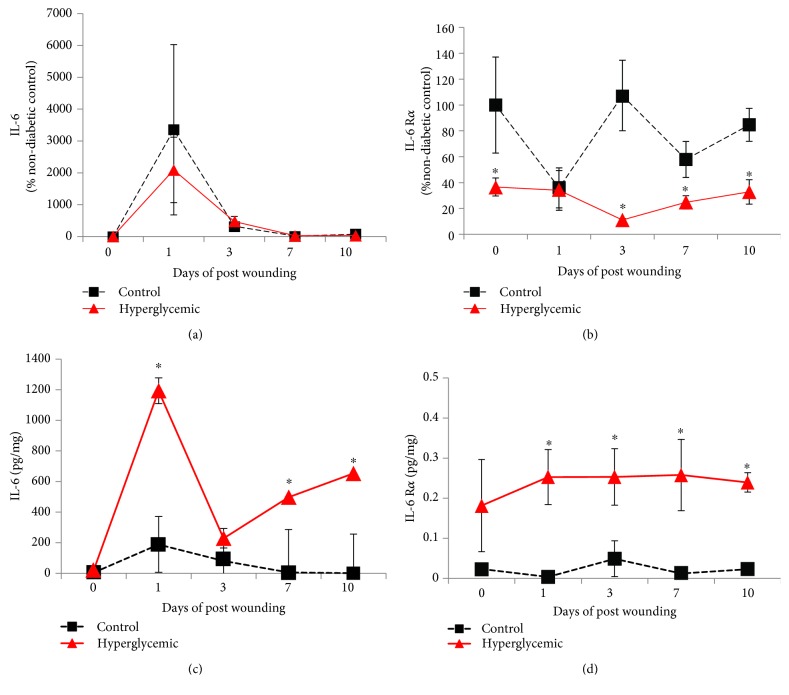
Hyperglycemic wounds exhibit greater IL-6 and IL-6R*α* protein expression. Following four weeks of hyperglycemia, hyperglycemic and age-matched euglycemic control mice were wounded with full-thickness 4 mm biopsy. Wound tissue was collected, and total mRNA and protein extract was isolated as described. (a) IL-6 and (b) IL-6R*α* mRNA expression was determined via qPCR and expressed as percent control (day 0 control animals). (c) IL-6 and (d) IL-6R*α* protein expression was determined by ELISA and normalized to total protein. Data are presented as the means ± SE; *n* = 10. ^∗^*p* < 0.05, significantly different from euglycemic C57BL/6 control mice.

IL-6 is a pleiotropic cytokine associated with the inflammatory and tissue remodeling phases of wound healing. IL-6-deficient mice display an early phase delay in wound healing [22, 23] similar to that observed herein with hyperglycemic mice, and IL-6 dysregulation is a well-known hallmark of diabetes. Thus, IL-6 and IL-6R*α* expression was assessed in wounds from hyperglycemic animals. IL-6 mRNA was transiently increased at day 1 postwounding and was not different between groups (Figure 2(a)). However, IL-6R*α* mRNA was significantly higher in control mice at all time points examined except day 1 (Figure 2(b)). As opposed to mRNA, wound IL-6 protein expression was significantly (6-fold) greater in hyperglycemic animals at days 1, 7, and 10 (Figure 2(c)). Likewise, IL-6R*α* protein expression was greater with similar dynamics on days 1, 3, 7, and 10 in hyperglycemic animals (Figure 2(d)).

### 3.3. Hyperglycemia Alters STAT3 Activation and SOCS3 Expression in Wounds

**Figure 3:**
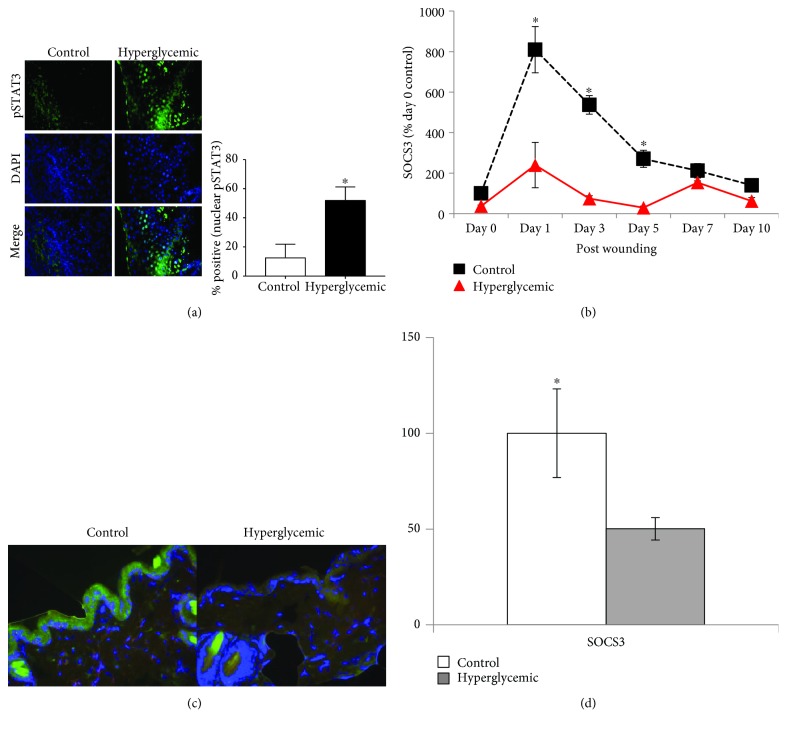
Hyperglycemia alters IL-6R signaling and function in the skin. Following four weeks of hyperglycemia, hyperglycemic and euglycemic control mice were wounded using a 4 mm biopsy. (a) Nuclear localization of pSTAT3 was determined in paraffin-embedded wound tissue by image analysis of immunohistology utilizing ImageJ64 software (v1.47, https://imagej.nih.gov/ij). Images were obtained at 400x magnification (*n* = 3). (b) SOCS3 mRNA expression in wound tissue analyzed via RT-PCR, and expression was expressed as percent control (day 0 control animals, *n* = 10). (c) SOCS3 expression in the epidermis was assessed by immunohistological staining (green), at 400x magnification, and (d) quantitated by ImageJ64 software as percent control value (*n* = 10). Data are presented as the means ± SE (^∗^*p* < 0.05, significantly different).

While IL-6 and IL-6R*α* expression seemed altered in hyperglycemic wounds, it was not apparent if this translated into altered function of the receptor. As STAT3 is well known to be phosphorylated by IL-6R stimulation, and indeed immunohistochemically staining and imaging analysis for pSTAT3 revealed significantly increased nuclear pSTAT3 levels in hyperglycemic 3-day wounds compared to controls (Figure 3(a)). SOCS3 is induced by IL-6R signaling and subsequent STAT3 activation in multiple cell types. Thus, to further assess the effects of diabetes on IL-6R-associated transcriptional activation, SOCS3 expression in the skin was assessed. SOCS3 mRNA expression in the skin is significantly decreased (up to ~3.5-fold) in hyperglycemic animals from days 1 to 3 of postinjury (Figure 3(b)). Immunohistochemical staining for SOCS3 in 3-day wounds was not different between groups (data not shown); however, staining of unwounded skin distal to the wound seemed to confirm SOCS3 mRNA data as there is nearly 2-fold decreased SOCS3 expression in hyperglycemic wounds (Figures 3(c) and 3(d)).

### 3.4. Hyperglycemia Alters IL-6R*α* Expression and IL-6 Induced STAT3 Activation in Primary Keratinocyte Cultures

**Figure 4:**
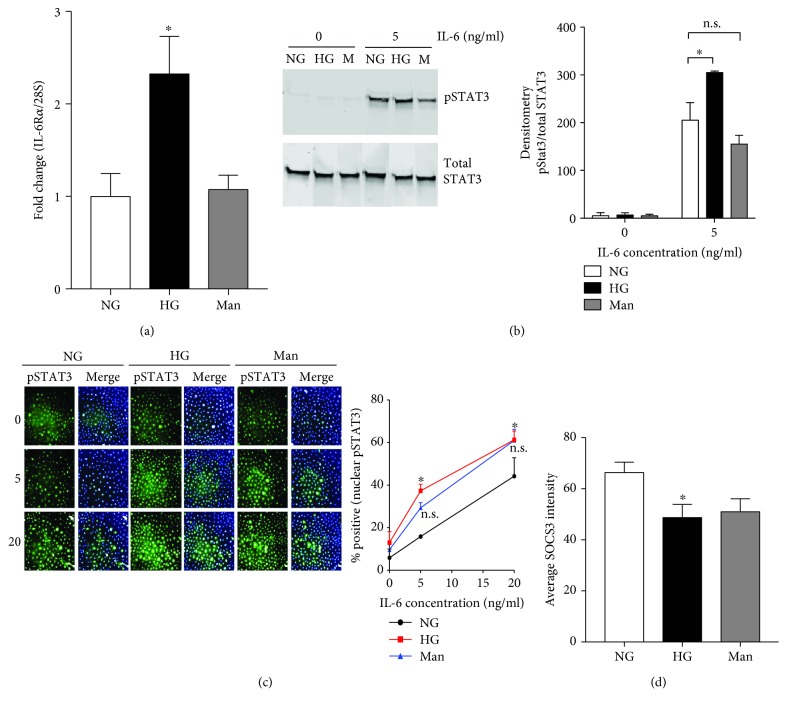
High glucose alters STAT3 activation in cultured keratinocytes. Neonatal mouse keratinocytes (MEKn) were cultured for 7 days in either normal glucose (NG; 5 mM), high glucose (HG; 25 mM), or mannitol osmotic control (Man; 5 mM glucose + 20 mM mannitol). (a) Basal IL-6R*α* mRNA expression was determined via qPCR. Data are presented as the means ± SD; *n* = 6 (3 replicates × 2 experiments). (b) MEKn were incubated with 0 or 5 ng/ml of rmIL-6 for 30 minutes. STAT3 phosphorylation (Y705) was determined via Western blotting (left panel) and quantified via densitometry analysis (right panel). (c) MEKn were treated with 0, 5, or 20 ng/ml of rmIL-6 for 30 minutes and stained for pSTAT3 (Y705) (green) and nucleus (blue). (d) MEKn were stained for SOCS3. Percentage of positive staining for pSTAT3 or SOCS3 was determined Operetta High-Content Imaging System (PerkinElmer, Waltham, MA) and analyzed with Columbus (v2.6.0, PerkinElmer). Data are presented as the means ± SD; *n* = 3. ^∗^*p* < 0.05 and ^∗∗^*p* < 0.01, significantly different from normal glucose-treated cells.

To determine if alterations in IL-6R*α* expression and pSTAT3 activation were directly associated with hyperglycemia, primary neonatal mouse epidermal keratinocytes (MEKn) were cultured in normal glucose (5 mM glucose), hyperglycemic (25 mM glucose), or mannitol (5 mM glucose + 20 mM mannitol) media for 7 days. Hyperglycemia induced increased basal IL-6R*α* mRNA expression as compared to normal glucose (NG) control (Figure 4(a)). To examine whether this expression is functional, MEKn cultured under hyperglycemic conditions for 7 days were treated with rmIL-6. Under hyperglycemic conditions, IL-6 induced an increase level of pSTAT3 as compared to normal glucose control while mannitol did not significantly affect pSTAT3 levels (Figure 4(b)). To assess the localization of pSTAT3, MEKn cultured under hyperglycemic conditions were assessed for IL-6-induced nuclear STAT3 phosphorylation via immunohistology staining. Staining for pSTAT3 was increased in MEKn cultured in hyperglycemic (25 mM glucose) conditions after stimulation with 5 and 20 ng/ml rmIL-6 as compared to normal glucose culture. Quantification of the percent of cells positive for nuclear pSTAT3 revealed a significant difference between cells in hyperglycemic conditions stimulated with 5 and 20 ng/ml rmIL-6 compared to normal glucose cells (Figure 4(c)). Basal levels of STAT3 were not affected by glucose concentration (Figure 4(b)). Basal SOCS3 levels in MEKn were as well examined, where the intensity of SOCS3 protein expression was significantly higher in 5 mM glucose as compared to 25 mM glucose culture (Figure 4(d)).

## 4. Discussion

A dysregulated inflammatory phase during diabetic wound healing is well known and variously linked to increased [39] and decreased [30] cytokine levels in the wound, seemingly depending on the model employed. However, levels of various inflammatory cytokines including TNF-*α* and IL-6 are increased in wound fluids from human diabetic ulcers [31]. IL-6 expression and signaling are crucial for normal wound healing, and indeed hyperglycemic mice display delayed wound healing remarkably similar to that observed in IL-6-deficient animals [22, 23] in this study (Figure 1). Diabetes is known to alter IL-6 expression as elevated serum IL-6 is reported in both type I and type II diabetes patients [40, 41]. Even so, most research concerning IL-6 and diabetes has focused on the role of IL-6 during the development and progression of diabetes. Given the crucial role of IL-6 in wound healing and its altered expression during diabetes, it was hypothesized that changes in IL-6 expression or signaling may be associated with delays in diabetic wound healing.

Data concerning the level of expression of IL-6 in diabetic wounds are conflicting, where an earlier report showed decreased expression [8] and another more recent showed increased mRNA expression 6 hours of postwounding [42]. In the present study, diabetic wounds from STZ-induced hyperglycemic mice showed greater IL-6 and IL-6R*α* protein (up to 6-fold) throughout the majority of the wound healing period (Figure 2). Interestingly, transcription of IL-6 and its receptor did not mirror protein levels. In fact, the effects are reversed in the case of the receptor where mRNA levels are consistently higher in wounds from control animals as compared to hyperglycemic (Figures 2(a) and 2(b)). This seems to indicate that the regulation of IL-6 and IL-6R*α* by hyperglycemia/diabetes resides at the posttranscriptional level.

The signaling function of the IL-6R complex is well described, with the canonical pathway leading to STAT3 activation. While IL-6R activates the Akt and MAPK [42, 43] pathways in dermal fibroblasts, STAT3 is known to be activated in keratinocytes, specifically at the wound edge, during healing as well as during skin pathologies such as psoriasis [44]. Indeed, it appears that increased expression of IL-6 and its receptor leads to a subsequent increase in phosphorylation of STAT3 at the hyperproliferative wound edge in hyperglycemic animals (Figure 3(a)). Paradoxically, expression of SOCS3 mRNA, which is well known to be directly upregulated by STAT3 activation, was significantly lower in hyperglycemic wound tissue (Figure 3(b)). While differences in SOCS3 protein expression (via immunohistology) at the wound edge were not different, indicating involvement of posttranscriptional regulation, it appeared that basal SOCS3 expression in the unwounded skin was significantly lower in hyperglycemic mice (Figure 3(c)). Aside from being induced by pSTAT3, SOCS3 is a direct inhibitor of gp130-mediated STAT3 activation [45], yet the role of SOCS3 in keratinocyte function and mitogenesis is complex. Keratinocytes themselves, cultured under normal conditions, appear to have an impaired ability to induce SOCS3 in response to IL-27, IL-6, and other cytokines as compared to macrophages (2- vs. 10-fold, respectively) [46]. When overexpressed specifically in the epithelium [47], SOCS3 decreased proliferation and migration of keratinocytes as a result of its inhibitory effect on STAT3 signaling. Indeed, delayed healing in *ob/ob* (leptin deficient) mice appears to be associated with increased SOCS3 expression in the skin [48]. However, specific deletion of SOCS3 in keratinocytes delayed healing as well, resulting in hyperproliferative epidermis and prolonged inflammation [49], where interestingly the two latter effects are hallmarks of diabetic wounds [50].

The exact mechanism of interplay between IL-6, STAT3, and SOCS3 at an organismal level during diabetes is likely complex; thus, it was of interest to determine if hyperglycemia itself might mediate altered IL-6R function in keratinocyte culture. Indeed, merely increasing hyperosmotic stress *in vitro* can increase IL-6 expression in epithelial cell lines [51]. While diabetes is a complex disease, and STAT3 can be phosphorylated by many cytokines that utilize the gp130 receptor [45], it appears that hyperglycemia (but not hyperosmolarity) is sufficient to increase IL-6-induced STAT3 phosphorylation in keratinocytes *in vitro* (Figures 4(b) and 4(c)). Further, similar to unwounded epithelium, SOCS3 expression in cultured keratinocytes is lower, not only under hyperglycemic but also hyperosmotic conditions (Figure 4(d)). This essentially parallels effects seen in cultured fibroblasts [52] where hyperosmotic stress potentiated LIF-induced pSTAT3 accumulation and concomitant reduction in SOCS3 expression. Yet, the current model indicated that osmotic stress was sufficient only to mediate decreased SOCS3 expression, while glucose was more potent than osmotic stress in potentiating IL-6R induced STAT3 activation. While the cause of this difference is not apparent, it may be associated with the level of osmotic stress where keratinocyte cultures herein were exposed to 20 mM excess glucose or mannitol, which is similar to unregulated diabetes (~450 mg/dl), versus 500 mM excess sorbitol [52].

## 5. Conclusions

As IL-6 is known to directly affect healing, particularly with respect to keratinocyte function [22, 23], these data seem to indicate a role for diabetic hyperglycemia in the alteration of IL-6R function in the epidermis. The apparent upregulation of IL-6 and its receptor in the diabetic skin as well as inhibition of SOCS3 may lead to increased STAT3 activation and ultimately result in altered keratinocyte function and/or dysregulated inflammation in the wound. However, as stated earlier, SOCS3 induction and STAT3 activation are not solely limited to IL-6 modulation and the diabetic wound is a complex immune environment. Thus, the mechanism by which gp130 function in keratinocytes might be modulated by diabetes or hyperglycemia is likely mediated by multiple cytokines and signal transduction crosstalk. Clearly, further research is required to elucidate the contribution and function of IL-6 in diabetic wound healing. Indeed, as the receptor and cytokine are overexpressed, it may be of great interest in the future to evaluate the effects of IL-6 agonism or antagonism for possible treatment evaluation, as well as specific keratinocyte knockout of IL-6R*α* (i.e., B6;SJL-IL6ra^tm1.1drew^/J) to further investigate mechanism.

## Data Availability

Data upon which all conclusions are drawn are included in the figures contained within this manuscript.
